# Closure of refractory gastrocutaneous fistula using submucosal dissection and over-the-scope clip assisted by a standard clip

**DOI:** 10.1055/a-2541-1960

**Published:** 2025-03-03

**Authors:** Eukene Rojo, Simona Agazzi, Mamadou Diakite, Pierre Lafeuille, Florian Rostain, Jérôme Rivory, Mathieu Pioche

**Affiliations:** 1 Gastroenterology, La Princesa University Hospital, Madrid, Spain; 2 18631 Gastroenterology and Digestive Endoscopy Unit, Fondazione IRCCS Policlinico San Matteo, Pavia, Italy; 3 Gastroenterology, University Hospital Center of Bouaké, Bouaké, Côte dʼIvoire; 4 Gastroenterology and Endoscopy Unit, Edouard Herriot Hospital, Hospices Civils de Lyon, Lyon, France

Percutaneous endoscopic gastrostomy is performed to gain enteral nutritional access in patients with impaired swallowing. However, gastrocutaneous fistula (GCF) may persist in up to 25% of cases following tube removal 11 . Endoscopic submucosal dissection (ESD) to re-epithelialize the fistulous tract, combined with closure using an over-the-scope (OTS) clip, has been reported as a safe and effective approach for managing persistent GCF 22 33 44 . A twin grasper is commonly used to pull the GCF into the OTS clip before the clip is deployed. A recently introduced, mantis-like claw clip – Mantis clip (Boston Scientific, Marlborough, Massachusetts, USA) – has demonstrated excellent efficacy in closing large gastrointestinal defects 55 . This reopenable, rotatable, through-the-scope clip features anchor prongs designed to securely grasp wound edges. Here, we describe a case in which we combined ESD of the GCF with closure using an OTS clip assisted by the Mantis clip, eliminating the need for a twin grasper and reducing costs.

We report the case of a 77-year-old patient with refractory GCF persisting 24 weeks after gastrostomy removal. A circumferential ESD was performed to ablate the fistulous tract and promote healing. Closure was achieved using an OTS clip (Ovesco, Olympus, Tokyo, Japan) assisted by a Mantis clip. Each edge of the fistula was sequentially grasped using the Mantis clip with a “hold-and-drag” strategy, preventing slippage. The OTS clip was then deployed, and contrast opacification confirmed the absence of leakage ( Video 1Video 1 ). The patient was discharged on post-procedure day 1 without complications and showed no recurrence at the 1-month follow-up.

Endoscopic submucosal dissection and closure of a gastrocutaneous fistula using a Mantis clip and an over-the-scope clip following percutaneous gastrostomy tube removal in a 77-year-old patient.Video 1Video 1

The design of the Mantis clip, featuring grasping teeth, provides excellent tissue grip and can serve as an alternative to the twin grasper when placing an OTS clip. Its greater availability and significantly lower cost compared to the twin grasper make this strategy particularly appealing.

Endoscopy_UCTN_Code_TTT_1AO_2AK

## Notes

Endoscopy E-Videos https://eref.thieme.de/e-videos
*E-Videos* is an open access online section of the journal *Endoscopy* , reporting on interesting cases and new techniques in gastroenterological endoscopy. All papers include a high-quality video and are published with a Creative Commons CC-BY license. Endoscopy E-Videos qualify for HINARI discounts and waivers and eligibility is automatically checked during the submission process. We grant 100% waivers to articles whose corresponding authors are based in Group A countries and 50% waivers to those who are based in Group B countries as classified by Research4Life (see: https://www.research4life.org/access/eligibility/ ). This section has its own submission website at https://mc.manuscriptcentral.com/e-videos .

## References

[1] Hucl T Spicak J Complications of percutaneous endoscopic gastrostomy Best Pract Res Clin Gastroenterol 2016 30 769 781 10.1016/j.bpg.2016.10.002 27931635

[2] Lafeuille P Wallenhorst T Lupu A Endoscopic submucosal dissection combined with clip for closure of gastrointestinal fistulas including those refractory to previous therapy Endoscopy 2022 54 700 705 34500487 10.1055/a-1641-7938

[3] Teitelbaum JE Gorcey SA Fox VL Combined endoscopic cautery and clip closure of chronic gastrocutaneous fistulas Gastrointest Endosc 2005 62 432 435 10.1016/j.gie.2005.04.047 16111964

[4] Gay-Chevallier S Lupu A Rivory J Closure of non-healing gastrocutaneous fistula after percutaneous endoscopic gastrostomy by endoscopic submucosal dissection and over-the-scope clip Endoscopy 2019 51 E125 E126 30866016 10.1055/a-0858-9796

[5] Nishiyama N Matsui T Nakatani K Novel strategy of hold-and-drag clip closure with mantis-like claw for post-gastric endoscopic submucosal dissection defect of < 30 mm Endoscopy 2023 55 E1244 E1245 38128588 10.1055/a-2213-4313 PMC10898239

